# Robotic Resection of Parapharyngeal Follicular Dendritic Cell Sarcoma With Castleman Disease

**DOI:** 10.1002/lary.70130

**Published:** 2025-09-15

**Authors:** Lily Huang, Aman M. Patel, Timothy Chao, Ryan M. Carey

**Affiliations:** ^1^ Perelman School of Medicine, University of Pennsylvania Philadelphia Pennsylvania USA; ^2^ Department of Otolaryngology–Head and Neck Surgery Rutgers New Jersey Medical School Newark New Jersey USA; ^3^ Department of Pathology and Laboratory Medicine University of Pennsylvania Philadelphia Pennsylvania USA; ^4^ Department of Otolaryngology–Head and Neck Surgery University of Pennsylvania Philadelphia Pennsylvania USA; ^5^ Department of Otolaryngology–Head and Neck Surgery Corporal Michael J. Crescenz Veterans Affairs Medical Center Philadelphia Pennsylvania USA

**Keywords:** Castleman disease, follicular dendritic cell sarcoma, minimally invasive surgery, parapharyngeal space

## Abstract

Parapharyngeal follicular dendritic cell sarcoma (FDCS) is rare and may occur with Castleman disease. We describe the successful transoral robotic resection of a parapharyngeal FDCS associated with Castleman disease, highlighting the role of the robotic approach in achieving tissue diagnosis and definitive treatment.

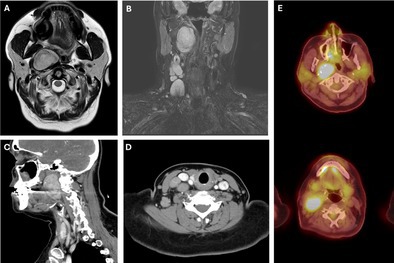

## 1 Introduction

Follicular dendritic cell sarcoma (FDCS) is a rare neoplasm of antigen‐presenting cells that typically involves cervical, axillary, and supraclavicular lymph nodes. Head and neck cases have been reported in the pharynx, palate, thyroid, and most commonly the tonsils. Approximately 10%–20% of FDCS cases are associated with Castleman disease (CD), especially the hyaline‐vascular subtype. FDCS is hypothesized to arise in the context of increased dendritic cell proliferation seen in CD. Both FDCS and CD may express epidermal growth factor receptor (EGFR), further suggesting a pathogenic link.

Only 16 cases of FDCS in the parapharyngeal space have been previously reported, none with associated CD [1, 2, 3, 4]. FDCS linked with CD has been described in the oral cavity and nasopharynx. Chan et al. [5] demonstrated progression of follicular dendritic cell proliferation in the nasopharynx in a patient with hyaline‐vascular CD. Historically, these parapharyngeal FDCS have been managed with invasive approaches such as mandibular split or mandibulectomy. To our knowledge, this is the first report of FDCS arising in the parapharyngeal space in the setting of CD, managed via transoral robotic surgery (TORS).

## 2 Case History

**FIGURE 1:**
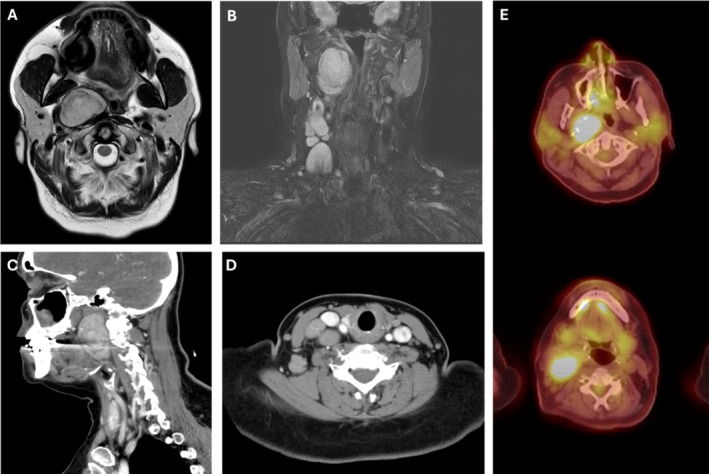
(A) Initial axial and (B) coronal T2 weighted MRI revealing a 4.0 cm mass in the right parapharyngeal space with ipsilateral lymphadenopathy. (C) Subsequent sagittal and (D) axial CT with contrast showing interval growth of the paraphyangeal space mass to 5.0 cm and stable lymph nodes. (E) Axial PET scan with FDG‐avid right‐sided parapharyngeal mass and lymph nodes. [Color figure can be viewed in the online issue, which is available at www.laryngoscope.com]

A 55‐year‐old woman with no significant medical history presented with acute right neck swelling and mild dyspnea. Flexible laryngoscopy showed nonobstructive bulging of the right lateral pharyngeal wall. Imaging revealed a 5 cm parapharyngeal mass with ipsilateral cervical lymphadenopathy (Figure 1A–D). Fine needle aspiration and core biopsy were non‐diagnostic, demonstrating reactive interfollicular hyperplasia with rare follicles. She was treated with steroids, resulting in near resolution of lymphadenopathy, though cough persisted.

She was referred for further evaluation. Exam revealed persistent parapharyngeal fullness and tender lymphadenopathy. She reported ear fullness, muffled hearing, and neck pain. Augmentin was started for possible infectious etiology with an equivocal response. Thyroid evaluation was negative. She was lost to follow‐up after being offered an excisional biopsy.

Five months later, she returned with worsening symptoms. Excisional biopsy of a level 3 node showed benign, reactive features. Infectious disease and rheumatology workups were negative. A positron emission tomography/computed tomography (PET/CT) scan revealed an FDG‐avid parapharyngeal mass and adjacent lymphadenopathy (Figure 1E).

Given the worsening symptoms and lack of definitive diagnosis, the patient was offered TORS resection of the parapharyngeal space mass which was the most PET‐avid region. The tumor was adherent to the skull base, prevertebral fascia, and ascending pharyngeal artery but was resected robotically without complication. She was discharged on postoperative day 5.

Evaluation by pathology demonstrated a 5 cm follicular dendritic cell sarcoma arising in the setting of CD. Multidisciplinary consultations recommended neck dissection for removal of pathologic nodes. Right neck dissection was subsequently performed, showing 32 nodes all negative for tumor or sarcoma but all consistent with unicentric CD. This contrasted with the initial excisional lymph node biopsy with a normal pathologic diagnosis.

The patient did well postoperatively and was referred to experts in Castleman disease, sarcoma medical oncology, and radiation oncology. Given close margins, she received proton beam therapy (60 Gy in 30 fractions) to the parapharyngeal site. Elective neck irradiation was deferred due to the absence of nodal sarcoma.

## 3 Pathologic Findings

**FIGURE 2:**
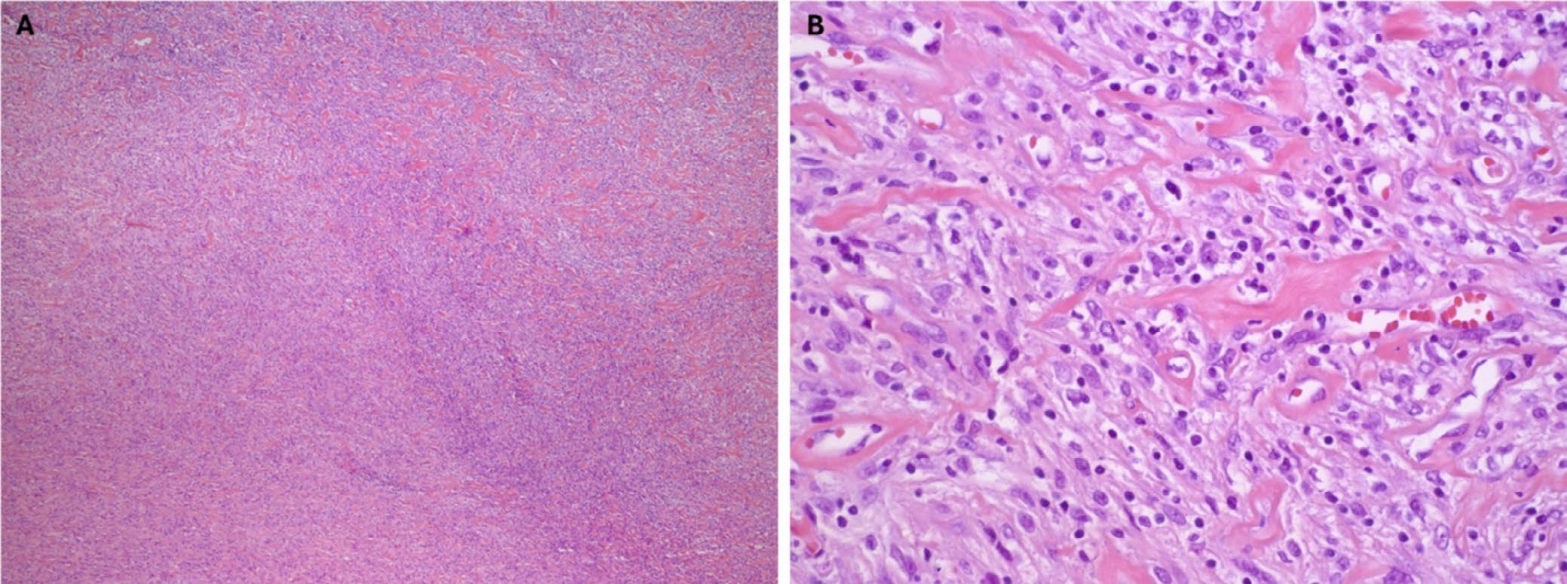
(A) Hematoxylin and eosin (H&E)‐stained sections showed portions of soft tissue with a diffuse infiltrate of atypical large cells with irregular nuclei, vesicular chromatin, conspicuous nucleoli, and moderate amounts of cytoplasm (original magnification 5×). (B) H&E‐stained sections show atypical cells are interspersed with small mature appearing lymphocytes (original magnification 40×). [Color figure can be viewed in the online issue, which is available at www.laryngoscope.com]

Histology revealed a diffuse infiltrate of atypical large cells with irregular nuclei, vesicular chromatin, conspicuous nucleoli, and moderate cytoplasm. These cells were interspersed with mature‐appearing lymphocytes (Figure 2A,B). Focal areas showed regressively transformed germinal centers and hyalinized vessels, consistent with CD.

**FIGURE 3:**
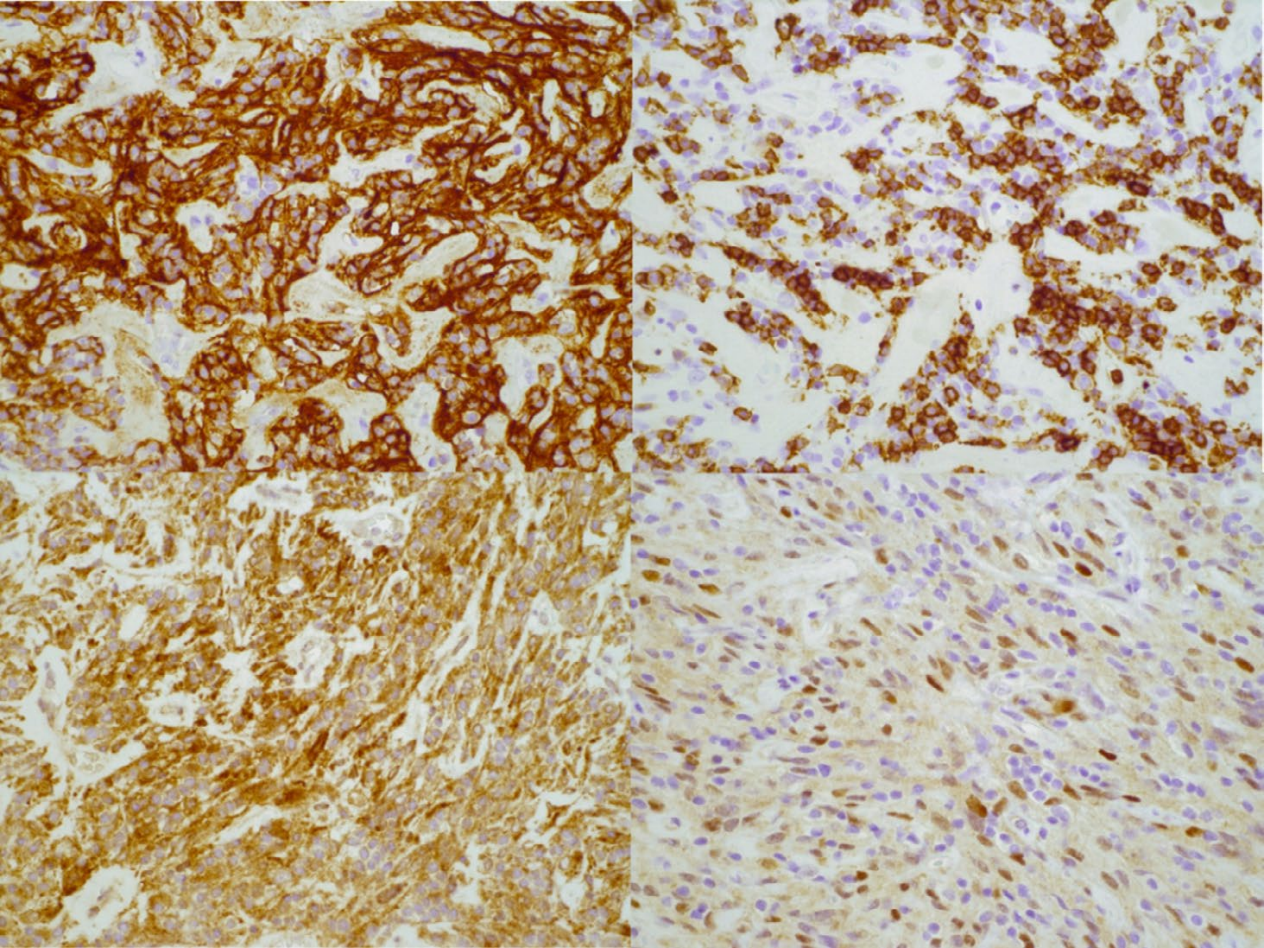
The neoplastic spindle cells were positive for CD21 (top left), CD23 (top right), D2‐40 (bottom left), and focally P63 (bottom right), (original magnification 40×). [Color figure can be viewed in the online issue, which is available at www.laryngoscope.com]

Immunohistochemistry showed tumor cells positive for CD21 and CD23 (strong), CD35 (weak), P63 (focal), and D2‐40 (Figure 3). Ki67 proliferation index was 5%–10%. Tumor cells were negative for CD34, TFE3, STAT6, ALK1, PAX8, AE1/3, and other epithelial, neural, and myogenic markers. CD3/CD5 highlighted small T‐cells; PAX5/CD20/CD21/CD23 showed scattered B‐cells.

## 4 Discussion

This is the first reported case of FDCS arising in the parapharyngeal space in the setting of CD, and the first managed with TORS. The case posed diagnostic challenges due to non‐diagnostic biopsies and a broad differential including infectious, autoimmune, and metastatic etiologies.

FDCS typically presents as a slow‐growing mass. PET/CT can show focal intense uptake. Histopathology and immunohistochemistry are essential for diagnosis, with characteristic markers including CD21, CD23, CD35, podoplanin, and CXCL13. CD often shows regressive germinal centers, which may transition to tumor proliferation.

Our case posed a management dilemma, as optimal treatment for FDCS with CD is not established, especially in the head and neck, further complicated by the need for surgery to make the diagnosis. Had the diagnosis been established preoperatively, the tumor would have been staged as T2N0, stage IIIA. According to NCCN guidelines, treatment for this stage would include (1) surgical resection to achieve appropriate oncologic margins when functional outcomes are acceptable, or (2) consideration of neoadjuvant systemic therapy, radiation therapy, or both together followed by surgery. Despite the diagnosis and staging not being confirmed until after resection, the surgical approach chosen in this case aligned with one of the guideline‐recommended strategies. This case highlights both the diagnostic and therapeutic role of surgical intervention in diagnostically challenging head and neck cases. Current management strategies include a multimodal approach consisting of surgery and adjuvant radiotherapy or chemoradiotherapy [5]. In one reported case of head and neck FDCS associated with CD, primary site surgical excision and post‐operative radiation therapy were administered with sparing of the regional lymphatics. Nine months later, the patient presented with enlarged lymph nodes and received subsequent irradiation to the ipsilateral neck and remained disease free. Another group reported CD with follicular dendritic cell overgrowth in the nasopharynx managed with transpalatal resection without adjuvant radiation; symptoms recurred after 3 years, and biopsy revealed FDCS in the background of CD. The patient was treated with 3 monthly cycles of chemotherapy with cyclophosphamide, doxorubicin, vincristine, and prednisone (CHOP) and underwent a nasopharyngectomy [5]. Given the rarity of this cancer, the necessary surgical margins and management of the neck are not well established. Similarly, there is debate about the inclusion of radiation and the optimal modality (proton vs. conventional radiotherapy), dose, and fields (primary site vs. regional lymphatics).

Sixteen prior parapharyngeal FDCS cases have been reported, none associated with CD (Table 1) [1, 2, 3, 4]. Most of these cases required open approaches such as mandibular split or mandibulectomy. Management of these cases included surgical resection with and without adjuvant therapy, with variable outcomes and frequent recurrences. Surgical resection of parapharyngeal FDCS has historically included invasive approache. This case is the first to describe the successful use of TORS, offering a minimally invasive route for both diagnosis and treatment.

**TABLE 1:** Summary of 16 cases of extranodal FDCS of the parapharyngeal region reported in the literature.

Reference	Sex/age	Size (cm)	Treatment	Adjuvant therapy	Outcomes
Desai et al.	f/45	6 × 3 × 3	Surgery (mandibulectomy)	RT	Local recurrence (31 m)
Vargas et al.	f/54	6	Surgery (mandibular split, craniotomy, tracheostomy, parotidectomy)	RT, CRT	Local recurrence (6 m)
Chan et al.	f/40	7 × 3 × 2	Surgery	N/A	Local recurrence (12 m)
Satoh et al.	m/16	3 × 2.5	Surgery (unspecified tumor resection and tonsillectomy)	RT, CRT	NED (24 m)
Al Hussain et al.	m/22	4 × 3 × 1.5	Surgery	RT	NED (26 m)
Dominguez‐Malagon et al.	f/29	4.8	Surgery	RT	Regional recurrence (invasion of the prevertebral fascia and sympathetic chain, 12 m)
Dominguez‐Malagon et al.	m/26	N/A	Surgery	RT, CRT	Distant recurrence (lung metastasis, 36 m)
Hu et al.	f/64	6 × 4 × 3	Surgery	RT	DOD (7 m)
Pyo et al.	m/31	4.7 × 4.5 × 1.9	Surgery	RT	N/A
Li et al.	f/35	5	Surgery	N/A	Distant recurrence (lung metastasis, 2 m; DOD, 12 m)
Li et al.	f/28	6	Surgery	RT, CRT	Distant recurrence (lung metastasis, 12 m; alive with disease 22 m)
Alexander et al.	m/69	3	Surgery	N/A	Local recurrence (12 m)
Kim‐Orden et al.	m/40s	4.3 × 2.7 × 5.8	Surgery (transcervical resection with transoral partial glossectomy, palatectomy, and pharyngectomy with reconstruction)	RT, CRT (CHOP)	Local recurrence (6 weeks, underwent re‐resection and neck dissection; no recurrence at 13 m follow up)
Li et al.	m/22	3.2 × 2.1 × 4.5	Surgery (left tonsillectomy, followed by transoral endoscopy assisted resection (of left parapharyngeal mass) and transcervical resection (of left cervical mass))	N/A	NED
Din et al.	m/25	5.5 × 4.0	Tumor debulking and lymph node sampling	Unknown	Unknown
Din et al.	m/22	5.5 × 4.0	Tumor debulking and lymph node sampling	CRT	DOD (9 m)

Abbreviations: CHOP, Cyclophosphamide, doxorubicin, vincristine, and prednisone combination therapy; CRT, chemoradiotherapy; DOD, died of disease; N/A, not applicable; NED, no evidence of disease; RT, radiotherapy; TORS, transoral robotic surgery.

## 5 Conclusion

This case highlights the diagnostic complexity of FDCS arising in the setting of CD and demonstrates that TORS offers a safe, minimally invasive approach for managing parapharyngeal space tumors. Continued reporting of such cases is essential to guide optimal diagnostic and treatment strategies for this rare entity.

## Ethics Statement

According to the Institutional Review Board (IRB) at the Hospital of the University of Pennsylvania, this case report does not meet the definition of human subjects research requiring IRB review. This study was conducted in accordance with the Declaration of Helsinki 1975.

## Conflicts of Interest

The authors declare no conflicts of interest.

## Data Availability

The data that support the findings of this study are available from the corresponding author upon reasonable request.
